# In sheep undergoing general anaesthesia does inclusion of medetomidine result in hypoxaemia?

**DOI:** 10.18849/ve.v10i2.708

**Published:** 2025-05-06

**Authors:** Rachael Gregson

**Keywords:** anaesthesia, analgesia, hypoxaemia, medetomidine, pharmacology, sheep

## Abstract

**PICO question:**

In healthy adult female non-pregnant sheep undergoing general anaesthesia for research studies does the inclusion of intravenous medetomidine as part of the anaesthetic protocol cause hypoxaemia?

**Clinical bottom line:**

**Category of research:**

Treatment.

**Number and type of study designs reviewed:**

Four papers were available for critical appraisal. The quality of the evidence is weak. There were four experimental studies; three of which were cross-over studies and one study which was run in parallel with primary orthopaedic research. None of the studies were specifically focussed on the potential hypoxaemic effects of medetomidine.

**Strength of evidence:**

Weak.

**Outcomes reported:**

Sheep across all four studies developed hypoxaemia (indicated by arterial oxygen tension; either P_a_O_2_ < 80 mmHg/10 kPa when breathing room air, or a statistically significant fall in P_a_O_2_ compared with baseline, when breathing oxygen enriched gases), when medetomidine was administered intravenously and in combination with various drugs (i.e. midazolam, propofol, ketamine, halothane, and isoflurane). However, as the sheep were receiving various doses of medetomidine at various timepoints, different quantities of supplemental oxygen (if any), varying ventilatory management (two studies used mechanical ventilation and two studies allowed sheep to breathe spontaneously), and different agents were used to maintain anaesthesia, the clinical significance of the P_a_O_2_ values was difficult to assess.

**Conclusion:**

In clinically healthy (judged by clinical examination) adult female non-pregnant sheep undergoing general anaesthesia (characterised by placement of an endotracheal tube and/or the use of anaesthetic induction agents i.e. barbiturates, ketamine, propofol), the weak evidence presented here suggests that use of intravenous medetomidine can be expected to cause hypoxaemia. However, hypoxaemia is variable and its clinical effects can be lessened with anaesthetic techniques such as the provision of supplemental oxygen.

## How to apply this evidence in practice

The application of evidence into practice should take into account multiple factors, not limited to: individual clinical expertise, patient’s circumstances and owners’ values, country, location or clinic where you work, the individual case in front of you, the availability of therapies and resources.

Knowledge Summaries are a resource to help reinforce or inform decision-making. They do not override the responsibility or judgement of the practitioner to do what is best for the animal in their care.

## Clinical scenario

You are a Named Veterinary Surgeon for a facility which uses large animals. A group of sheep will be receiving general anaesthesia for detailed computed tomography (CT) scanning as part of a Home Office licensed project. The principal investigator is concerned that medetomidine, which is administered as part of the facility’s standard anaesthetic protocol, will cause hypoxaemia, and wants advice as to how relevant this may be.

## The evidence

Hypoxaemia develops in healthy adult female non-pregnant sheep following intravenous medetomidine administration as part of a general anaesthetic protocol. The quality of the evidence is weak; there are four experimental studies; three cross-over studies (Raekallio et al., 1998; Celly et al., 1999a; Raisis et al, 2021) and one study which was run in parallel with primary orthopaedic research (Kästner et al., 2001). All four studies varied as to their anaesthetic and ventilatory management of the sheep. Although the evidence shows that hypoxaemia occurs following the intravenous administration of medetomidine in sheep in the peri-anaesthetic period, the severity and clinical consequences of this hypoxaemia are difficult to determine. This is due to the variation in protocols between the studies. The evidence suggests that when intravenous medetomidine is administered to healthy adult female non-pregnant sheep hypoxaemia (partial pressure of arterial oxygen [P_a_O_2_] < 80 mmHg/10 kPa when breathing room air or a statistically significant fall in P_a_O_2 _from baseline when breathing oxygen enriched gases) develops. The drug’s effects on P_a_O_2_ are minimised by anaesthetic techniques such as the administration of supplemental oxygen, tracheal intubation, and adequate monitoring. Therefore, intravenous medetomidine could be used cautiously alongside appropriate anaesthetic management in healthy adult female non-pregnant sheep undergoing research procedures. However, medetomidine is not licensed in food-producing animals in the UK and adherence to relevant legislation in the country of use is advised.

## Summary of the evidence

**Celly et al. (1999a):** Cardiopulmonary Effects of the α2-Adrenoceptor Agonists Medetomidine and ST-91 in Anesthetized Sheep **Aim: **To investigate the relative contributions of the cardiovascular and respiratory systems to alpha2-agonist-induced hypoxemia in halothane anaesthetised, ventilated sheep by comparing the effects of incremental doses of the central and peripheral acting a2-agonist, medetomidine, the peripherally acting a2-agonist, ST-91, and a saline placebo.

Population	Healthy adult female non-pregnant Arcot ewes.
Sample Size	5 sheep.
Intervention Details	One month before experimentation—relocation of carotid artery subcutaneously to allow for blood sampling in this study. Each sheep was used three times, with at least 7 days wash-out between studies. Each sheep received each treatment; the order was randomised, but the method of randomisation was not described. Anaesthesia and instrumentation: Induction with pentobarbital sodium (20 mg/kg IV). Tracheal intubation. Inhalational maintenance with 1% halothane vaporised in oxygen as the sole carrier gas. Neuromuscular blockade with intravenous atracurium (0.2 mg/kg). Each dose was administered subsequently at approximately 25-minute intervals. Step-wise test drug administration with either: Medetomidine (0.5, 1.0, 2.0 and 4.0 µg/kg IV). ST-91 (1.5, 3.0, 6.0 and 12.0 µg/kg IV) (ST-91 is a peripherally acting alpha-2 agonist). Saline (placebo)—2 ml administered IV at each time point. Arterial catheterisation to allow for arterial blood pressure measurement and blood sampling. Jugular catheterisation to allow for placement of a Swan-Ganz thermistor catheter. Volume controlled mechanical ventilation to normocapnia.
Study design	Cross-over experimental study.
Outcome studied	Objective: Spirometry; dynamic compliance, total pulmonary resistance, transpulmonary pressure_._ Cardiovascular; mean arterial pressure, systemic vascular resistance, shunt fraction, cardiac index , pulmonary arterial pressure, pulmonary vascular resistance, pulmonary wedge arterial pressure. Respiratory; arterial and mixed venous oxygen tension, arterial carbon dioxide tension, alveolar-arterial oxygen tension gradient. Other; thromboxane.
Main findings (relevant to PICO question)	Statistically significant fall in arterial oxygen tension after administration of all doses of medetomidine in halothane anaesthetised sheep, compared to placebo (from 509 mmHg to 214 mmHg after the first dose of medetomidine [0.5 µg/kg], P < 05). Increase in alveolar-arterial oxygen tension gradient after all doses of medetomidine (P < 05). Results for the other treatment groups (administration of ST-91 or saline) are not included as they did not meet the PICO’s definition for anaesthesia.
Limitations	The sequential doses of medetomidine (approximately 25 minutes apart) would have had an additive/cumulative effect (half-life is approximately 30 minutes) allowing for potential drug accumulation. No power calculation. Method of randomisation not specified. Small sample size. Plasma concentrations of medetomidine or ST-91 were not measured. Results are presented graphically (not as a table) meaning individual values, such as means ­+ standard deviation, are difficult to ascertain unless stated in the text. Fate of experimental sheep not recorded.

Table note...

**Kästner et al. (2001):** Comparison of Medetomidine and Dexmedetomidine as Premedication in Isoflurane Anaesthesia for Orthopaedic Surgery in Domestic Sheep **Aim: **To determine the potency of dexmedetomidine in relation to medetomidine in sheep undergoing orthopaedic surgery by comparing the anaesthetic requirements and cardiovascular effects of equipotent doses of both drugs in healthy adult female sheep.

Population	Healthy adult non-pregnant female sheep, various breeds.
Sample Size	24 sheep.
Intervention Details	This study was run in parallel with an orthopaedic study (hip replacement) which will not be discussed further in this appraisal. Two treatment groups, 12 sheep in each group: Group 1—received 5 µg/kg dexmedetomidine intravenously as premedication. Group 2—received 10 µg/kg medetomidine intravenously as premedication. Both groups received their premedication 5 minutes prior to the induction of anaesthesia—after premedication, all sheep underwent the same anaesthesia and surgery consisting of: Jugular catheterisation. Induction with intravenous ketamine (2 mg/kg). Two sheep required diazepam to allow intubation and were excluded for further comparisons. Maintenance with isoflurane vaporised in 100% oxygen (FE’_ISO_ was 1.02 +04% [Group 1] and 0.99 + 0.07% [Group 2]). Lactated Ringer’s solution was administered at 10 ml/kg/hour via the jugular catheter. Sheep were allowed to breathe spontaneously. Auricular arterial cannulation for blood pressure measurement.
Study design	Blinded, randomised, experimental trial.
Outcome studied	Level of sedation. Ease of tracheal intubation. Surgical plane of anaesthesia. Duration of anaesthesia and of surgical procedure. Time to extubation, sternal recumbency and standing. Measured every 5 minutes during anaesthesia: Rectal temperature. Electrical activity of the heart and heart rate via electrocardiogram. Respiratory rate. Arterial blood pressure. Expired gases (carbon dioxide, isoflurane). Measured every 30 minutes during anaesthesia Arterial blood gases (pH, carbon dioxide, and oxygen).
Main findings (relevant to PICO question)	Group 1 did not receive medetomidine; therefore, their results are not discussed within this appraisal. In group 2 (receiving medetomidine), after an initial fall immediately after induction of anaesthesia to 58.5 mmHg, P_a_O_2_ increased significantly to 395.7 mmHg 10 minutes after induction, once supplemental oxygen was administered during anaesthesia. Body position did not affect blood gas variables.
Limitations	Method of blinding not described. Method of randomisation not described. Sedation not scored/quantified. Lack of a control group. Data was only recorded after induction of anaesthesia, which was approximately 15 minutes after the premedication. Fate of sheep not described. States in discussion that “pulmonary oedema” in individual animals is the cause of “severe hypoxaemia”, but there is no histological examination reported.

Table note...

**Raekallio et al. (1998):** Medetomidine-Midazolam Sedation in Sheep **Aim: **To investigate the effects (on sedation, arterial oxygenation and haemoglobin oxygen saturation) on healthy adult female sheep of a low dose of medetomidine combined with midazolam; the effects of this combination were compared to the effect of each drug when used alone.

Population	Healthy female non-pregnant landrace sheep, approximately one year old.
Sample Size	7 sheep.
Intervention Details	Historical right carotid translocation for previous study, which allowed for arterial blood sampling in this study. Each sheep was sedated three times, with at least 7 days wash-out between studies. Jugular catheterisation. Intravenous drug administration via catheter, either one of the following three treatments. Medetomidine (15 µg/kg). Midazolam (0.1 mg/kg). Medetomidine (15 µg/kg) immediately followed by midazolam (0.1 mg/kg). Trachea was intubated and arterial blood pressure was monitored (pre-sedation and at 5, 10, 20, 30, 40, 50, and 60 minutes post sedation) in the medetomidine/midazolam group only. If recumbent, sheep were placed in left lateral recumbency. Monitoring of heart rate and respiratory rate using electrocardiogram and thoracic auscultation/observation, respectively. Arterial blood samples (for oxygen and carbon dioxide tension [P_a_O_2_ and P_a_CO_2_]), pH, haemoglobin concentration ([Hb]), haemoglobin oxygen saturation (S_a_O_2_) and base excess) taken prior to sedation, and 5, 10, 20, 30, 40, 50, and 60 minutes thereafter.
Study design	Cross-over experimental study.
Outcome studied	Subjective: Level of sedation. Objective: Heart rate. Respiratory rate. Arterial oxygen and carbon dioxide tension, haemoglobin saturation, pH, base excess. Arterial blood pressure (medetomidine-midazolam group only).
Main findings (relevant to PICO question)	Medetomidine-midazolam caused hypoxaemia as shown by P_a_O_2_ (< 7 kPa [< 52.5 kPa]) and S_a_O_2_ (< 90%). This was significantly reduced from baseline values (P < 0.05). One sheep in this group suffered a cardiac arrest and required resuscitation twice. This combination of drugs (medetomidine and midazolam) was not recommended for use in sheep. Results for the other treatment groups (medetomidine alone or midazolam alone) are not included as they did not meet the PICO’s definition for anaesthesia.
Limitations	Small study numbers, no power calculation. Blinding and randomisation not described. Sedation not scored/quantified. Groups were not all treat similarly i.e. only the medetomidine-midazolam group had arterial blood pressure measurements taken and tracheal intubation performed. Results are only presented graphically (not as a table). Values such as the mean, standard deviation may be difficult to ascertain due to the presentation of the results. Fate of experimental sheep not recorded. Humane endpoints not recorded.

Table note...

**Raisis et al. (2021):** Comparison of pulmonary function in isoflurane anaesthetized ventilated sheep (Ovis aries) following administration of intravenous xylazine versus medetomidine **Aim: **To compare the effects of equipotent intravenous doses of two alpha-2 adrenoreceptor agonist drugs (xylazine and medetomidine) on pulmonary function (as assessed by spirometry, volumetric capnography and arterial blood gases) on isoflurane anaesthetised, mechanically ventilated, healthy adult female sheep.

Population	Healthy non-pregnant adult ewes (Merino and Greeline).
Sample Size	40 sheep.
Intervention Details	This study was run in parallel with a nutritional study which will not be discussed further in this appraisal. Each ewe received each of the two treatments, with a 40-day interval between them. Anaesthesia: Jugular venous catheterisation. Sedation with intravenous diazepam (0.2 mg/kg). Anaesthesia was induced with intravenous propofol (2 mg/kg). Tracheal intubation. Anaesthesia maintenance with isoflurane. Neither the end tidal isoflurane nor the inspired tension of oxygen were stated. Mechanical ventilation was imposed. Orogastric tube placed. CT scan of thorax. Drug administration with either Xylazine (intravenous) 75 µg/kg, subsequently reduced to 37.5 µg/kg after adverse effects (hypoxaemia). Sheep receiving higher doses were subsequently excluded from data analysis by the study author*.* Medetomidine (intravenous) 5 µg/kg, subsequently reduced to 2.5 µg/kg to maintain an equipotent dose comparable to xylazine. Sheep receiving higher doses were subsequently excluded from data analysis*.* These doses were considered equipotent (i.e. 75 µg/kg xylazine = 5 µg/kg medetomidine, and 37.5 µg/kg xylazine = 2.5 µg/kg medetomidine). Atipamazole (intramuscular) 0.3 mg/kg given to reverse xylazine/medetomidine if haemoglobin oxygen saturation fell to below 90% or tachypnoea non-responsive to ^f_R_ occurred; data subsequently excluded if atipamezole administered.
Study design	Randomised, cross-over experimental study.
Outcome studied	Recorded at T0, T5, and T10 following drug administration (T0—immediately prior to injection with medetomidine, T5 and T10 are 5 and 10 minutes following injection with medetomidine, respectively): Spirometry; respiratory frequency, tidal volume, expired minute ventilation, mean airway pressures, peak inspiratory pressures, peak expiratory flow, airway resistance, dynamic system compliance. Volumetric capnography; end-tidal carbon dioxide elimination/breath and /minute, alveolar minute ventilation, airway dead space/tidal volume ratio. Recorded at T0 and T10; samples taken via percutaneous puncture: Arterial oxygen tension (also used to calculate arterial/inspired (P_a_O_2_/F*_I_*O_2_) ratio). Arterial carbon dioxide tension.
Main findings (relevant to PICO question)	Intravenous premedication with medetomidine in Merino and Greeline ewes caused: decreases in P_a_O_2 _from T0 (63.1 kPa) and T10 (56.1 kPa) time points (P < 0.05) although never reaching clinical hypoxaemia i.e. remains < 10 kPa. decreases in P_a_O_2_/F*_I_*O_2_ ratio between T0 (483 minutes) and T10 (420 minutes) time points (P < 0.05) although P_a_O_2_/F*_I_*O_2_ ratio did remain >300, i.e. didn’t meet ARDS definition. No sheep required atipamezole administration.
Limitations	No power calculation, sample size dictated by concurrent study. No dose-finding pilot could be performed, meaning doses of the drugs in question had to be altered once the study had commenced; doses had been extrapolated from a study using conscious sheep. F*_I_*O_2_ not specified during anaesthesia phase, although used to calculate PaO_2_/F*_I_*O_2_ Measurements were not able to be taken during the first 5 minutes following medetomidine administration, when maximal effects may have been present, due to CT scanning Exact timing of medetomidine or xylazine administration is not stated.

Table note...

## Appraisal, application and reflection

Sheep are commonly used as experimental research animals for translational biomedical research, particularly orthopaedic and neurological research due to “its large body size, gyrencephalic brain, long lifespan, more extended gestation period, and similarities in neuroanatomical structures to humans” (Banstola & Reynolds, 2022). The provision of balanced anaesthetic protocols which provide analgesia are therefore required in these procedures. Alpha-2 adrenoreceptor agonist drugs (hereafter referred to as alpha-2 agonists) such as medetomidine, have analgesic, sedative, and anaesthetic sparing effects and are very useful as part of balanced anaesthetic techniques in sheep (Lizarraga & Chambers, 2012). Alpha-2 agonists are known to cause hypoxaemia in sheep but there is variation between individual animals and the degree of hypoxaemia is dependent upon dose and route of administration; the causal mechanism is proposed to be alpha-2 adrenoreceptor (hereafter called “α2”) mediated pulmonary vasoconstriction and bronchoconstriction leading to alveolar oedema (Kästner, 2006). Medetomidine is the most specific alpha-2 agonist with an alpha-2:alpha-1 adrenoreceptor selectivity ratio of 1620:1 (Dugdale, et al., 2020) meaning that side effects due to alpha-1 receptor stimulation are less likely to occur than when less α2 specific drugs, such as xylazine, are used. This Knowledge Summary therefore aims to investigate if treatment with intravenous medetomidine in healthy adult non-pregnant sheep causes hypoxaemia, defined as P_a_O_2_ < 80 mmHg/< 10 kPa when breathing room air, or a statistically significant fall from baseline when supplemental oxygen is administered.

A literature review was performed to answer the PICO question, which yielded four peer-reviewed papers for critical appraisal, after applying article exclusion criteria (Raekallio et al., 1998; Celly et al., 1999a; Kästner et al., 2001; Raisis et al., 2021). All the studies are experimental; three were cross-over studies (Raekallio et al., 1998; Celly et al., 1999a; Raisis et al., 2021) and one was run in parallel with an orthopaedic study (Kästner et al., 2001). There was blinding of evaluators to treatment in only one of the studies (Kästner et al., 2001), and randomisation in only three of the studies (Celly et al., 1999a; Kästner et al., 2001 and Raisis et al, 2021). The articles retrieved covered a time period, from 1998 to 2021.

Synergism between alpha-2 agonists and other anaesthetic drugs is well recognised. Chemical immobilisation using large doses of medetomidine (0.125 mg/kg) and ketamine (2.5 mg/kg) has been shown to cause significant hypoxaemia (P_a_O_2 _< 40 mmHg) in sheep during the initial phases of immobilisation (Caulkett et al., 1994). However, the authors of this study suggest this could be mitigated with supplemental oxygen administration and reversal of the medetomidine with atipamezole, although this may render the chemical immobilisation ineffective.

Celly et al. (1999a) found hypoxaemia while increasing doses of medetomidine (0.5 µg/kg to 4.0 µg/kg, intravenously) were administered to mechanically ventilated sheep anaesthetised with halothane (Celly et al. 1999a). The alveolar-arterial oxygen tension gradient (P(A-a)O_2_) and shunt fraction (Q_s_/Q_t_) increased, signifying pulmonary dysfunction in these animals, which is offered as the explanation for the fall in arterial oxygen tension (P_a_O_2_). The pulmonary dysfunction is suggested to be caused by the development of pulmonary oedema which has been shown to occur after xylazine administration (Celly et al., 1999b). As both xylazine and medetomidine are alpha-2 agonists with a comparable mode of action, it is reasonable to agree with the authors that the development of pulmonary oedema could also be occurring here. However, none of the papers discussed here described evidence of pulmonary oedema. The P_a_O_2 _in the region of 200 mmHg following medetomidine administration in these sheep is in excess of the P_a_O_2_ of approximately 100 mmHg a healthy animal breathing room air would achieve, according to the alveolar gas equation [P_A_O_2_ = F*_I_*O_2_(P_B_-P_H2O_) - (P_A_CO_2_/RQ)]; Dugdale et al., 2020. However, as the sheep in this study were breathing 100% oxygen (Celly et al., 1999a), and their P_a_O_2_ was 509 mmHg when the placebo was administered, the sheep receiving medetomidine can be considered to have a relative hypoxaemia, the effects of which were lessened by oxygen supplementation; this fall in P_a_O_2_ was statistically significant (P < 0.05).

Hypoxaemia was found, with the P_a_O_2_ reaching a lowest level of 58.5 mmHg, in sheep premedicated with medetomidine (10 µg/kg intravenously) during isoflurane anaesthesia for experimental hip replacement surgery (Kästner et al., 2001). However, it is difficult to assess these numbers as the F*_I_*O_2_ was not stated, the sheep were breathing spontaneously, and their respiratory function under anaesthesia was sub-optimal, as indicated by a raised P_a_CO_2_ in the region of 50 mmHg; normal being 35–45 mmHg (Dugdale et al., 2020). This paper did not include a negative control group for ethical reasons. It is therefore difficult to prove that the hypoxaemia is due to the alpha-2 agonist, and not due to another reason, such as effects of recumbency or surgery. Also, the paper concludes with the statement that there is “development of severe hypoxaemia and pulmonary oedema in individual animals” yet no individual animal data is included, only ranges, and no histology was performed to confirm the presence of pulmonary oedema or fat emboli (Kästner et al., 2001); fat emboli can develop after bone surgery and cause hypoxaemia, and these sheep had had hip replacement surgery (Lindeque et al., 1987).

The most recent paper yielded by the literature search was Raisis et al., (2021). This considered respiratory function in isoflurane anaesthetised, mechanically ventilated sheep administered either xylazine or medetomidine (2.5–5 µg/kg intravenously). Like the paper by Kästner et al. (2001), this was performed concurrently with another experimental study, in this case a nutritional study, and as such was restricted in terms of timeline, numbers and endpoints by the primary study. No dose finding pilot to choose appropriate doses of alpha-2 agonist drugs could therefore be performed, and the alpha-2 agonist doses had been extrapolated from a study using conscious sheep (Celly, et al., 1997). The methodology of the study was altered on discovery that the chosen initial dose of intravenous xylazine (75 µg/kg) caused significant hypoxaemia (assessed as SpO_2_ < 90% or marked tachypnoea and confirmed by a P_a_O_2_ of 51.2 [37.4–64.9] mmHg) necessitating reversal of the xylazine with atipamezole. The dose of both alpha-2 agonists was subsequently halved to maintain equipotent doses and the data from the sheep given higher doses was excluded. No sheep receiving medetomidine required administration of atipamezole. The data from the animals eventually included in the paper shows that the sheep receiving medetomidine in comparison with xylazine did experience a fall in P_a_O_2_, from 63.1 kPa (473.3 mmHg) before drug administration to 56.1 kPa (420.8 mmHg) at 10 minutes after drug administration (P < 0.05). Again, the animals are reported to be mechanically ventilated with 100% oxygen at this point, mitigating the effects of any pulmonary dysfunction caused by the medetomidine, as shown by the increase in carbon dioxide elimination (VCO_2_). The study concludes with the statement that medetomidine is preferred over xylazine as it better maintains pulmonary function but suggests that more work is required in other sheep breeds.

In a cross-over experimental study (Raekallio et al., 1998), sedation in sheep was investigated using medetomidine, midazolam, and a combination of the two agents; only the combination of medetomidine and midazolam met the criteria for general anaesthesia in this Knowledge Summary (allowing tracheal intubation). This study showed that 15 µg/kg medetomidine and 0.1 mg/kg midazolam administered intravenously caused profound sedation leading to lateral recumbency accompanied by marked hypoxaemia (on the basis of P_a_O_2_ measurement) unrelated to ventilation (assessed by P_a_CO_2_ values). However, although there are graphs of the P_a_O_2_ values over time, due to the granular nature of the scale and lack of numerical table of values, it is difficult to determine exactly the level of hypoxaemia in these sheep, although it speculated to be in the region of 5–7 kPa (37.5–52.5 mmHg). As the definition of clinical hypoxaemia for the purposes of this Knowledge Summary is P_a_O_2_ < 80 mmHg or < 10 kPa when breathing room air (Dugdale et al., 2020), it is possible that if these sheep received supplemental oxygen, and perhaps if the lungs had been mechanically ventilated, which would have been possible as their tracheas had been intubated, their P_a_O_2_ would not have fallen to this level. One sheep in this group suffered cardiac arrest and had to be resuscitated; although out with the remit of this Knowledge Summary to discuss the ethics of the experimental studies appraised, it raises the topic of humane end points for this study. The main conclusion of the paper is that the synergism between midazolam and medetomidine causes such profound cardiorespiratory effects that their use cannot be recommended.

All the papers discussed here found that hypoxaemia, as assessed by P_a_O_2_, occurs after the administration of intravenous medetomidine in the peri-anaesthetic period in healthy adult female non-pregnant sheep of various breeds. The severity and clinical significance is, however, not easy to determine, as none of the studies was primarily interested in the development of hypoxaemia following the administration of intravenous medetomidine. There was a wide variation in doses of medetomidine administered, ventilatory support provided and levels of oxygen supplementation administered, meaning the hypoxaemia identified may not have been relevant in a clinical situation. The evidence, although weak, suggests that in answer to the PICO question, intravenous medetomidine administered to adult female sheep judged healthy on clinical exam during the anaesthetic period causes hypoxaemia.

## Methodology

**Search Strategy:** Caption placeholder

Databases searched and dates covered	CAB Abstracts (via CABI Digital Library): 1900–June 2024 PubMed (via NIH National Library of Medicine): 1900–June 2024 Web of Science (via Institute for Scientific Information): 1900–June 2024
Search terms	CAB Abstracts: sheep OR ovine (Article Title) AND anaesthe* OR anesthe* (Abstract) AND hypoxaemia OR hypoxemia (Abstract) AND medetomidine (Abstract) PubMed: (("sheep" OR "ovine") AND ("anaesthe*" OR "anesthe*") AND ("hypoxaemia" OR "hypoxemia") AND (medetomidine)) Web of Science: sheep OR ovine (Title) AND anaesthe* OR anesthe* (Topic) AND hypoxaemia OR hypoxemia (Topic) AND medetomidine (Topic)
Dates searches performed	14 Jun 2024

Table note placeholder

**Exclusion/Inclusion Criteria:** Caption placeholder

Exclusion	Not available in electronic format from the University of Liverpool library. Sheep not anaesthetised (no endotracheal tube placed and/or induction agent i.e. barbiturate, ketamine, propofol, not administered). Medetomidine not administered. P_a_O_2_ not reported. Review article.
Inclusion	Describes healthy adult female non-pregnant domestic sheep undergoing anaesthesia (i.e. placement of an endotracheal tube and/or administration of an induction agent i.e. barbiturate, ketamine, propofol) where medetomidine is administered.

Table note placeholder

**Search Outcome:** Caption placeholder

Database	Number of results	Excluded— wrong species	Excluded — sheep not anaesthetised	Excluded — unavailable via the University of Liverpool Library	Excluded — medetomidine not administered	Excluded— review article	Total relevant papers
CAB Abstracts	8	2	1	2	0	2	1
PubMed	10	3	0	1	0	2	4
Web of Science	13	2	2	2	2	2	3
Total relevant papers when duplicates removed							4

Table note placeholder

### Acknowledgements

The author would like to acknowledge that this Knowledge Summary was initially conceived as part of a Certificate of Advanced Veterinary Practice (CertAVP) module studied at the University of Liverpool.

### ORCiD

Rachael Gregson: https://orcid.org/0000-0003-2704-1825

## Conflict of Interest

The authors declare no conflicts of interest.

## Disclaimer

Knowledge Summaries are a peer-reviewed article type which aims to answer a clinical question based on the best available current evidence. It does not override the responsibility of the practitioner. Informed decisions should be made by considering such factors as individual clinical expertise and judgement along with patient’s circumstances and owners’ values. Knowledge Summaries are a resource to help inform and any opinions expressed within the Knowledge Summaries are the author’s own and do not necessarily reflect the view of the RCVS Knowledge. Authors are responsible for the accuracy of the content. While the Editor and Publisher believe that all content herein are in accord with current recommendations and practice at the time of publication, they accept no legal responsibility for any errors or omissions, and make no warranty, express or implied, with respect to material contained within. For further information please refer to our Terms of Use.
